# Effect of Oxygen-Containing Group on the Catalytic Performance of Zn/C Catalyst for Acetylene Acetoxylation

**DOI:** 10.3390/nano11051174

**Published:** 2021-04-29

**Authors:** Fulong Zhu, Junqing Li, Mingyuan Zhu, Lihua Kang

**Affiliations:** 1 College of Chemistry & Chemical Engineering, Yantai University, Yantai 264010, China; zhufulong1994@126.com (F.Z.); zhuminyuan@shzu.edu.cn (M.Z.); 2 School of Chemistry and Chemical Engineering, Shihezi University, Shihezi 832003, China; lijunqing@stu.shzu.edu.cn

**Keywords:** oxygen-containing groups, dicarboxylic catalytic system, density functional theory

## Abstract

In this study, a series of activated carbon-based supports with different oxygen-containing groups (OCGs) proportions were obtained via thermal treatment in an ozone flow. Semiquantitative analyses indicated that the performance of the catalyst attained a maximum after 30 min of treatment with ozone flow, and had a positive correlation with the content ratios of carboxyl and hydroxyl groups. Further, temperature-programmed desorption analysis demonstrated that the high performance (63% acetic acid conversion) of the prepared catalyst for the acetoxylation of acetylene could be ascribed to the reduced strength of increased capacity of acetylene adsorption. Density functional theory proved that the additional –COOH in the dicarboxylic catalytic system could be employed as a support for the active sites, and enhancing C_2_H_2_ adsorption strength in the rate-limiting step in the actual experimental process effectively accelerated the reaction rate. Thus, the OCGs on the surface of activated carbon play a crucial role in the catalytic performance of the acetylene acetoxylation catalyst.

## 1. Introduction

As one of the extensively applied chemical intermediates, vinyl acetate (VAC) is widely used in numerous chemical processes [1,2]. Presently, the main methods of synthesizing VAC are the acetoxylations of acetylene [3] and ethylene [4]. Therein, zinc acetate is impregnated and adsorbed onto activated carbon (AC), which is a catalyst that has been utilized industrially for the acetylene acetoxylation reaction [5]. The possible reaction mechanism with zinc acetate as the active site for acetylene acetoxylation has been well investigated [6,7]. First, acetic acid is absorbed by zinc acetate to form a complex. Thereafter, VAC is produced by the reaction of the absorbed acetylene molecule with the formed complex. Regarding this mechanism, the rate-limiting step is the adsorption of acetylene [6]. Some scholars [7,8,9] have demonstrated that the adsorption strength and capacity of acetylene significantly influences the acetylene acetoxylation reaction. Thus, the regulation of the adsorption of acetylene must be considered in the field of catalysis science. Thus far, various studies on the supports and catalysts of the acetylene acetoxylation reaction, including N [9,10] and B-doped ACs [11] as the support and metal additives [8], have been developed through the density functional theory (DFT) investigation [7]. Further, heteroatom-doping via the introduction of heteroatoms such as B [11] and N [9,10], has been employed to change the surface electronic structure of AC and adjust the interaction among the different atoms inside, changing the adsorption capability of the catalyst and improving the conversion rate of acetic acid. Dong et al. [7] calculated the bond energy between zinc acetate and different oxygen-containing groups (OCGs) and estimated the complexity of the reaction that occurred in the active sites (zinc acetate that was adsorbed on the hydroxyl (–OH) and carboxyl (–COOH)). The roles of different OCGs have been revealed superficially by demonstrating that the reaction barriers of the rate-limiting step in this case was reduced by the existence of –COOH. Although many scholars achieved slightly positive results on the acetoxylation of acetylene, the following series of challenges still exist: the low conversion of acetic acid and the mechanization of OCGs [8,9]; the slightly higher OCGs contents cause the low utilization of the zinc component [10] and the DFT results of OCGs have not been effectively supported experimentally [7]. Whether the specific influence of OCGs on the acetoxylation of acetylene is due to the interaction of OCGs with zinc, electron transfer, or other phenomena such as the adsorption capacity, which strongly impacts the catalytic performance of the catalyst, there are no experimental data and characterization measures available. Thereby, some relevant studies on the acetoxylation of acetylene must be considered.

It is well-known that many OCGs, such as the –COOH, –OH, carbonyl (–C=O), and epoxide (C–O–C) groups, are widely distributed on the surfaces of all kinds of carbon materials [12,13,14,15,16,17,18]. These materials have been utilized as supports or metal-free catalysts in many reactions, such as hydrogenation and CO_2_ reduction reactions [19,20,21]. OCGs on the surface of carbon can synergistically catalyze its reaction with metal components and effectively strengthen its adsorption capability for different reactants, thus sequentially facilitating the high performance of the metal catalyst [22,23]. OCGs in the carbon matrix can induce local charge redistributions, thereby improving the binding affinity between the activators and the carbon atoms [17]. The electron-rich O centers of oxygen-doped carbon materials were identified as the active sites for some reactions [18]. Therefore, the essential role of OCGs in catalysis has been broadly revealed. Bai et al. [13] grafted–COOH onto the surface of graphitic carbon nitride, and the electron-withdrawing effect boosted the adsorption and separation of the reactants, thereby enhancing the performance of the catalyst. Han et al. [15] utilized ultraviolet ozone (UV-O_3_) to regulate the impact of OCGs in porous carbon spheres at different processing times. They changed the loading conditions of the metal component and enhanced the adsorptions of different reactants, increasing the activity of the catalyst. De La Puente et al. [24] confirmed that the rate of the complexation of a metal component was increased by adding OCGs. Many –COOH groups increased the adsorption of the metal component and reactants. Tan et al. [25] implanted OCGs in an Rh/AC catalyst that was treated by HNO_3_ at different temperatures. The influence of OCGs in the catalyst on the reaction was clarified for the first time by this catalyst. Additionally, OCGs in the catalyst optimized its surface properties, thus affecting the reaction and adsorptions of the reactants onto the active sites. Chang et al. [26] pretreated a catalyst, carbon black, with ozone and supported it on platinum, and the performance of the catalyst was investigated. During the treatment, more –COOH functional groups were detected on the surface of carbon black, and they functioned as additional adsorption sites. Additionally, structural damage and reductions in surface areas were not observed in these materials, indicating that the deterioration of the durability and activity of the catalyst was avoided.

Based on the discrepancies in the interactions of different OCGs with metal components and the adsorptions of different reactants, the proportions of different OCGs on the surface of AC were regulated by ozone pretreatment under heating conditions at different processing times. A series of experiments and characterization techniques were used to elucidate the idiographic functions of OCGs in the acetylene acetoxylation reaction. X-ray photoelectron spectroscopy (XPS) analysis [21] and the Boehm titration method [27] were employed to investigate the correlation of the different OCGs contents on the surface of AC during the ozone treatments. Further, additional experiments and characterization analyses were employed to evaluate the performance of the prepared catalysts in the acetoxylation of acetylene. The effect of OCGs on the performance of the catalyst was revealed by exploring its adsorption capacity and strength for different reactants. The establishment of different adsorption models and the DFT measurement further deepened our understanding of the role of OCGs in the acetoxylation of acetylene.

## 2. Materials and Methods

### 2.1. Chemicals 

Deionized water (18.2 Mohm·cm^−1^, Purelab Option Q, ELGA, Bukinghamshire, UK) was utilized as the solvent. Ozone was prepared by a FeiLi Ozone-oxygen machine (30% (volume), 2 L·min^−1^, FL-810ET, Shenzhen, China). The materials utilized included AC (coconut shell carbon), C_2_H_2_ (99.99%, Weichuang gas, Shanghai, China), CH_3_COOH (99.5%, Yongsheng, Tianjin, China), and zinc acetate (99.8%, Aldrich, Shanghai, China).

### 2.2. Preparation of Carbon Supports

Synthesis of ozone treated AC (AC-x): AC-x was obtained at different ozone treatment durations (x = 10, 20, 30, 45, and 60 min). First, 5 g of AC was placed in a three-necked round-bottom flask and heated to 413 K in a thermostatic oil bath. When the temperature reached 413 K, an ozone flow (30%, 2 L·min^−^^1^) was introduced for x min, then switched to nitrogen (60 mL·min^−1^, 1 h) and the sample cooled to room temperature. All the samples obtained were named AC-x to clearly distinguish between the non-OCGs ACs and the other ACs (AC-x).

### 2.3. Preparation of Catalysts

All the catalysts were prepared by the impregnation and filtration methods. Typically, 2 g of zinc acetate and 20 mL of deionized water were stirred for 1 h at 298 K. Subsequently, 2 g of the prepared AC-x was added and stirred for 12 h at 298 K, then the obtained mixture was washed three times with deionized water and subjected to vacuum filtration before it was dried for 10 h at 353 K. These catalysts obtained were named Zn/AC-x (x = 10, 20, 30, 45, and 60).

### 2.4. Catalytic Performance Test

The performance of each of the above catalysts for the acetoxylation of acetylene was evaluated by a fixed-bed reactor (12 mm stainless steel tube). Briefly, 1.2 g of the catalyst was added to the reactor and heated to 453 K in a nitrogen atmosphere. Subsequently, 0.02 mL·min^−1^ of CH_3_COOH (l), which was passed through the gasification equipment at 423 K, was added to the reactor to activate the catalyst, after which the reaction temperature was adjusted to 493 K. After 30 min, the nitrogen gas flow was changed to pure C_2_H_2_ (20 mL·min^−1^). The obtained products were analyzed by a Shimadzu GC-9A gas chromatograph equipped with a dual-channel N2000 chromatography data station.

### 2.5. Catalysts Characterization

The Brunauer-Emmett-Teller (BET) analysis was conducted on a Micromeritics ASAP 2460 apparatus at 77 K and temperature-programmed desorption (TPD) measurements were conducted by heating the catalysts from 353 to 1073 K at a 10 K·min^−^^1^ in an argon atmosphere. Furthermore, XPS was performed on Thermo Scientific K-Alpha equipment. The zinc content of the catalysts was measured by inductively coupled plasma (ICP) evaluation employing the iCAP 6000 series ICP emission spectrometer. The Fourier-transform infrared (FT-IR) spectra of all the supports were obtained on a Thermo Scientific Nicolet IS10 instrument. The KBr disk method was employed with a scan range of 4000–400 cm^−1^. Scanning electron micrograph (SEM) characterization was performed using a Zeiss Sigma 300 field-emitting microscope with a 15 kV accelerating voltage.

## 3. Results and Discussion

### 3.1. Texture Properties of AC-x

**Figure 1:**
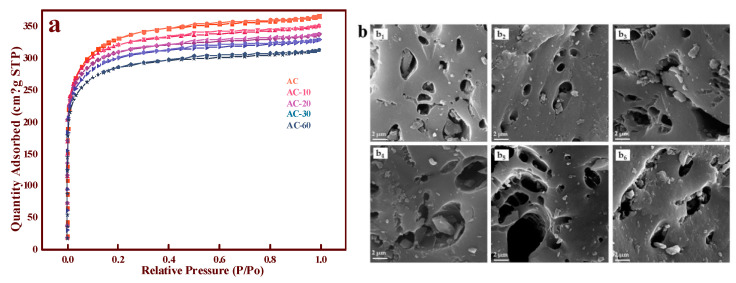
(**a**) Nitrogen adsorption-desorption isotherms of AC and AC-x with different ozone treatment times; (**b**) SEM images of different support: AC (b_1_), AC-10 (b_2_), AC-20 (b_3_), AC-30 (b_4_), AC-45 (b_5_), AC-60 (b_6_).

The nitrogen adsorption-desorption isotherms of AC, AC-10, AC-20, AC-30, and AC-60 are shown in Figure 1a. According to the IUPAC classification [13], all the samples exhibited IV isotherms with an H4 hysteresis loop, illustrating that microporous and mesoporous structures existed in all the samples. However, we observed that the BET surface area decreased from 1083 (AC) to 922.7 m^2^·g^−1^ (AC-60), and the pore volume diminished as the ozone treatment time increased, as presented in Table 1. The discrepancies in the different samples were not significant, and the diameters of all the samples were similar. These results indicate that the surface modifications did not significantly damage the basic skeleton of AC and that there were no redundant ions or groups in the samples [14]. The SEM picture, meanwhile, was a strong complement to this result, and significant differences could not be observed on all the samples shown in Figure 1b. Therefore, after eliminating the possible impacts of the structural changes, we suspected that the surface OCGs exerted a major influence on the catalytic performance.

### 3.2. Analysis of OCGs on the Surface

**Figure 2:**
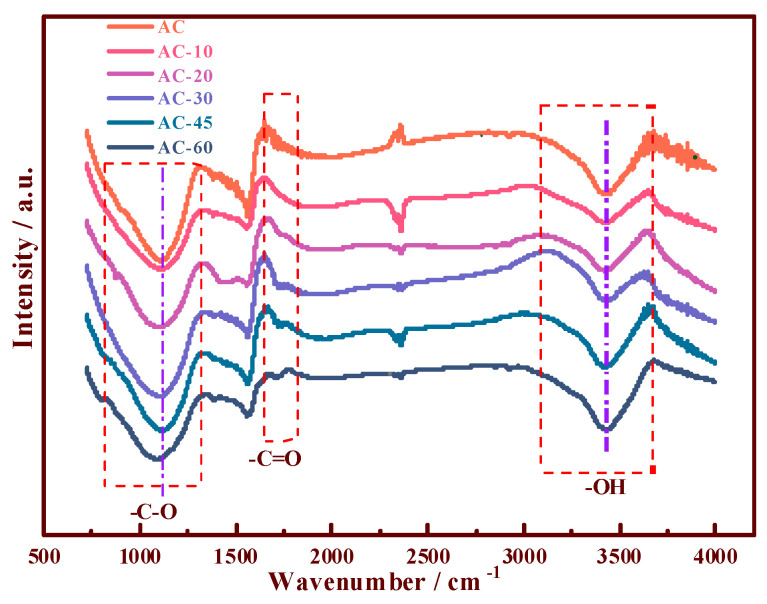
FTIR spectra of the support samples of the prepared AC and ozone-pretreated AC-10, AC-20, AC-30, AC-45, and AC-60.

Based on the FTIR characterizations (Figure 2), the compositions of the different OCGs were analyzed. Compared with the spectrum of the traditional AC, the contribution of the peak at 1716 cm^−1^ [14,21] increased, indicating the formation of new –C=O groups (O–C–O or –COOH) in the different materials (AC-20, AC-30, AC-45, and AC-60) during processing. Moreover, –OH and –C–O, which consisted of –COOH, were observed at 3400 and 1100 cm^−1^, respectively. The –OH and –C–O groups were observed in the 750–1250 and 3200–3700 cm^−1^ regions. All the samples demonstrated that more OCGs, especially –COOH, were introduced to the surface of AC. To further reveal the changes in OCGs on the supports before and after the ozone treatment, the Boehm titration method (Appendix A) [27] and XPS [28,29] analysis were exploited to investigate the property of the AC surface.

**Figure 3:**
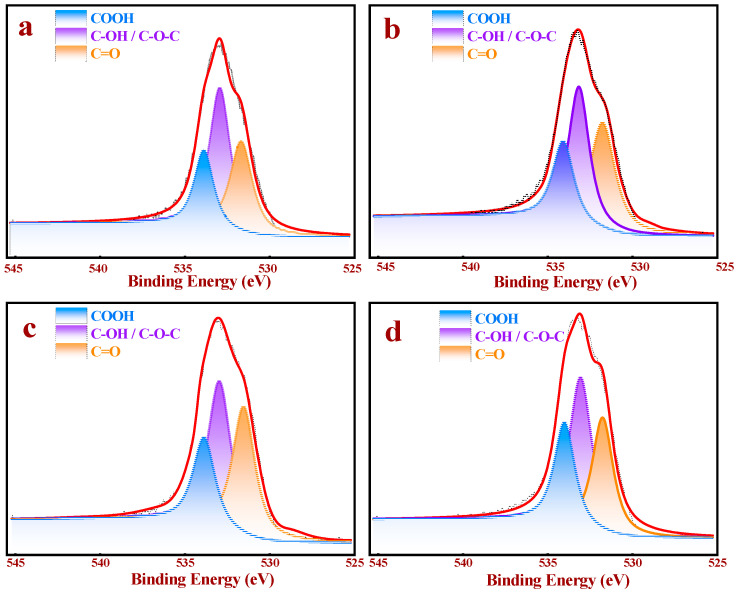
XPS O 1s spectra of the different supports: (**a**) AC, (**b**) AC-10, (**c**) AC-30, and (**d**) AC-60.

As presented in Table 2 and Figure 3 regarding the Boehm titration method, the total oxygen content of AC was 5.10%. However, as the ozone treatment durations increased, the total oxygen content also increased to 11.37% in AC-60, indicating that the ozone treatment had effectively enhanced the surface oxygen contents of AC. After 10 min of treatment, the –OH content did not significantly change (from 1.58% to 1.84%), but was enhanced conspicuously with the continuous increase in the processing time (from 1.58% (AC) to 4.23% (AC-60)). Regarding the –COOH contents, which were determined by the Boehm titration method, they distinctly increased after the treatment for 10 min, reaching the maximum value (6.48%) after 30 min of treatment when the positive effects of –COOH reached the optimal value. In contrast, the negative effects of –OH increased with increasing ozone treatment durations. Therefore, the performance of the Zn/AC-30 catalyst improved greatly, while those of the Zn/AC-45 and Zn/AC-60 catalysts decreased slightly.

To deeply understand the roles of the OCGs content on the surface of the samples, XPS analysis, which was applied to prove the rationality of the Boehm titration method, was employed to quantify the surface chemical compositions of AC and AC-x, as shown in Figure 3 and Table 2. The O 1s spectrum in Figure 3 could be divided into three peaks –COOH (531.5 eV), –C–OH/–C–O–C (532.9 eV), and –C=O (533.9 eV) [30], respectively.

Employing these results, the proportions of the different OCGs on the surfaces of AC and AC-x were calculated (Table 2). The total oxygen contents of all the samples increased continuously by increasing the duration. Regarding –COOH, there was a significant increase (from 1.15% (AC) to 2.57% (AC-30)), although the –COOH contents were only 2.46% after 60 min of the ozone treatment. These results proved that the –COOH contents of all the ozone-treated samples achieved their maximum values after 30 min of treatment. According to Table 2, the –OH content demonstrated continuous growth (from 2.37% to 4.84%), which corresponds to the Boehm titration results. The results demonstrated that the different OCGs contents of AC, AC-10, AC-30 and AC-60 exerted different and significant effects on the performances of the catalysts. Therefore, we evaluated the influences of different OCGs proportions on the acetoxylation of acetylene by experiments, characterization techniques and DFT.

### 3.3. Catalyst Testing

**Figure 4:**
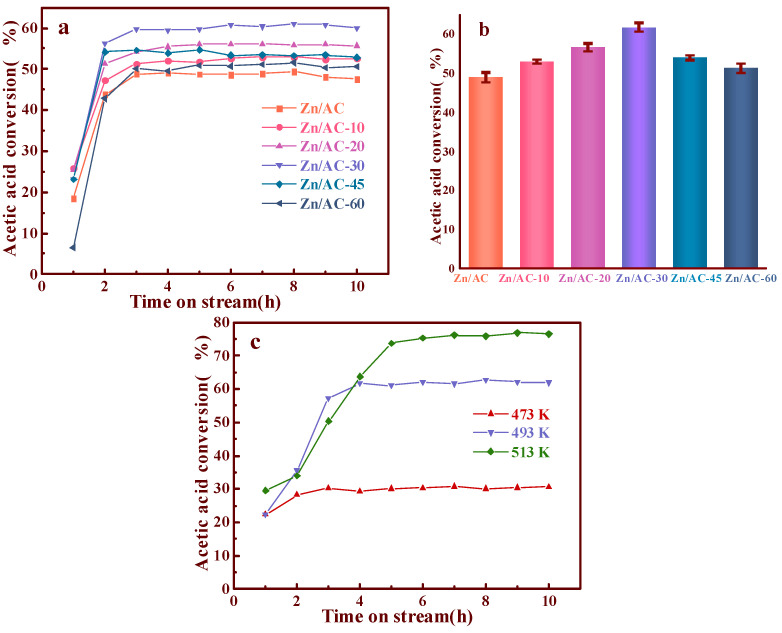
Conversion of CH_3_COOH catalyzed by Zn/AC and Zn/AC-x (**a**); error bars analysis of Zn/AC and Zn/AC-x catalysts (**b**); the effect of reaction temperature (**c**).

Via ICP measurement, all the catalysts possessed similar Zn contents (7.1% by mass), as determined by the ICP measurements. It is possible to ascribe the different performances (Figure 4) of the Zn/AC-x catalysts to OCGs on the surface of the different supports. As shown in Figure 4, there was a huge disparity in the performance of the ozone-treated AC as supports at different durations. Compared with untreated AC, the performance of the catalysts gradually increased after treatments of 10, 20, and 30 min. The treated AC achieved its maximum value (63% CH_3_COOH conversion rate) concurrently with Zn/AC-30. However, Zn/AC-45 and Zn/AC-60 exhibited slight decreases in their performances. To validate results, error bars were measured for the catalysts, as shown in the Figure 4b. These results add rigor to the experiment.

The effect of reaction temperature was investigated in our experiment. As shown in Figure 4c, we found that the acetic acid conversion was only 30% at 473K. Although the conversion of acetic acid reached 76% at 513 K, the thermal decomposition of zinc acetate at 513 K is a major limitation [31]. Therefore, we chose 493 K as the reaction temperature.

### 3.4. The Relation of Catalytic Performance with the Contents of Different OCGs

Combining the FTIR analysis results and the performance evaluations (Figure 4) of all the catalysts, we suspected that we had successfully regulated OCGs of AC and employed ozone to increase the –COOH content, which was significant to the acetoxylation of acetylene.

**Figure 5:**
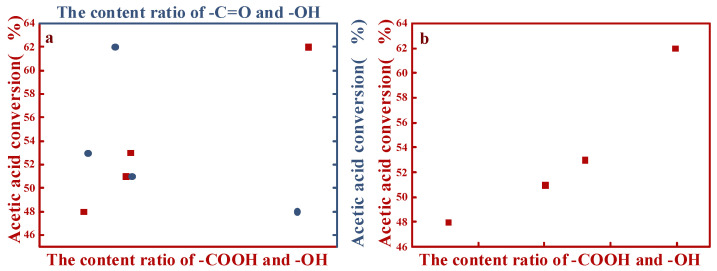
Analyses of the relationships of the catalytic performances of different catalysts with different OCGs content ratios: (**a**) XPS and (**b**) Boehm titration.

Related to the Boehm titration and XPS data, a correlation diagram of the performances of the catalysts and different OCGs content ratios on the surface of AC was obtained (Figure 5). Regarding the XPS analysis (Figure 5a), the conversion of acetic acid was directly proportional to the content ratios of –COOH and –OH (red spots); more acetic acid was transformed into VAC as the ratio increased. However, regarding the –C=O and –OH content ratios (blue spots), there was barely a correlation between their ratios and the performances of the different catalysts. The –COOH and –OH content ratios were also obtained by Boehm titration. The performances of the different catalysts exhibited positive correlations with the –COOH and –OH content ratios (Figure 5b). Thus, both results supported the positive correlations of the catalysts with the surface –COOH contents and their negative correlations with –OH on the AC surface [7].

### 3.5. Study on the Association between Zn and Different OCGs

**Figure 6:**
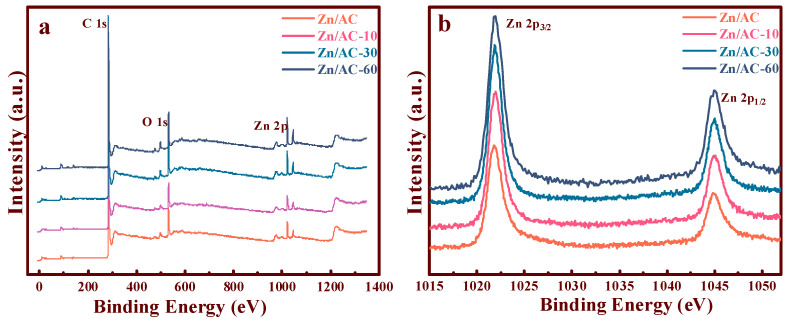
XPS spectra of the synthesized Zn/AC-x catalysts: (**a**) wide survey spectra; (**b**) Zn 2p region.

To explore the concealed relation between the active sites with OCGs, the XPS spectra (Figure 6a) distinctly exhibited the Zn 2p, O 1s, and C 1s signals of the Zn/AC, Zn/AC-10, Zn/AC-30, and Zn/AC-60 catalysts. Moreover, Figure 4b illustrates the energy spectra of the Zn 2p orbitals of the Zn/AC, Zn/AC-10, Zn/AC-30, and Zn/AC-60 catalysts. The Zn 2p_3/2_ and 2p_1/2_ peak positions of all the catalysts were clearly observed at 1021.8 and 1045.03 eV, respectively [9,32]. Thus, the binding energies of Zn 2p with different catalysts did not significantly deviate, and the introduction of OCGs did not change the electron cloud density of zinc significantly.

### 3.6. The Effect of OCGs for the Catalysts

**Figure 7:**
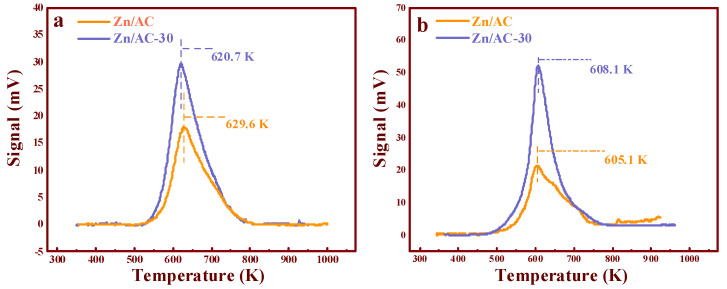
TPD analysis of different reactants: (**a**) C_2_H_2_ and (**b**) CH_3_COOH.

TPD analysis was employed to further understand the reasons for the high performances of the catalysts (TPD details: Appendix B) [11]. As shown in Figure 7a, the adsorption capacity of C_2_H_2_ by the catalysts changed after the ozone treatment compared with that of Zn/AC. Further, the desorption temperature of C_2_H_2_ distinctly reduced from 629.6 K (the Zn/AC catalyst) to 620.7 K (the Zn/AC-30 catalyst). Based on our previous studies [11], the decrease in the desorption temperature played a positive role in the acetoxylation of acetylene. Furthermore, the desorption area of the Zn/AC-30 catalyst was much larger than that of the Zn/AC catalyst, i.e., the adsorption capacity of C_2_H_2_ by the catalyst was apparently enhanced after the ozone treatment for 30 min. Compared with Zn/AC, Zn/AC-30 obtained the highest desorption temperature (608.1 K) for adsorbing CH_3_COOH, indicating that the adsorption strength of CH_3_COOH was slightly enhanced. Previous studies proved that the adsorption of C_2_H_2_ in the acetoxylation of acetylene is potentially the rate-limiting step [6]. Thus, the increase in C_2_H_2_ adsorption capacity promoted the progress of the reaction. However, an excessively high C_2_H_2_ adsorption strength would not favor the proper functioning of the active center, while a low adsorption strength would not favor the rate-limiting step. This implied that the high performance of the Zn/AC-30 catalyst was mainly ascribed to the high adsorption capacity and an appropriate adsorption strength. Moreover, the enhanced adsorption of CH_3_COOH also impacted the performances of the catalysts significantly, as demonstrated by a previous study [11], and implied that the supernumerary –COOH group could promote the absorption capacity of C_2_H_2_ on the active sites post ozone treatment. It is also possible that additional –COOH groups could change the adsorption strength of C_2_H_2_ within the reaction duration. As many scholars have demonstrated, –COOH could be utilized as an accelerant for the adsorption of many reactants and organics [13,15,24,25,26], and it exerted a significant impact on the performance of the catalyst. To deeply understand the intrinsic role of dicarboxylic AC, the adsorption energies of C_2_H_2_ on different structures were studied by DFT.

### 3.7. DFT Calculation

**Figure 8:**
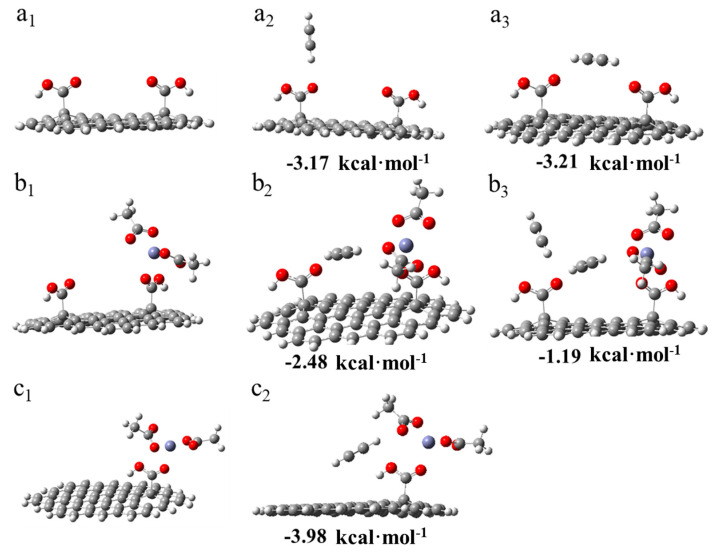
DFT results of the adsorptions of the acetylene molecule. (**a_1_**) Dicarboxylic substrate of AC. (**a_2_**) Adsorption of the acetylene molecule on the first –COOH group of AC with the dicarboxylic substrate (AC–II–COOH*). (**a_3_**) Adsorption of the acetylene molecule on the middle of AC with AC–II–COOH*. (**b_1_**) Adsorption of zinc acetate on dicarboxylic AC. (**b_2_**) First adsorption of the acetylene molecule between the –COOH group and another molecule, which was adsorbed zinc acetate. (**b_3_**) Adsorption of the second acetylene molecule. (**c_1_**) Adsorption of zinc acetate on monocarboxylic AC. (**c_2_**) Adsorption of acetylene on the monocarboxylic catalyst.

Two adsorption models of C_2_H_2_ on dicarboxylic AC were simulated, as shown in Figure 8(a_2_,a_3_). The adsorption energy of a single acetylene molecule on the first –COOH group was −3.17 kcal·mol^−1^. Another modeled condition was the adsorption of the acetylene molecule on the middle of the dicarboxylic groups, and the adsorption energy was >−3.21 kcal·mol^−1^. The two results indicate that the C_2_H_2_ molecule could be adsorbed on the –COOH groups, thus indicating that the –COOH groups were potential adsorption sites. Thereafter, the models of the adsorption of zinc acetate on the dicarboxylic (b_1_) and monocarboxylic groups (c_1_) were established. The adsorption energy of the first C_2_H_2_ molecule was −3.98 kcal·mol^−1^ when it was adsorbed on the monocarboxylic catalytic system (c_2_). By contrast, the adsorption energy was −2.48 kcal·mol^−1^ when it was adsorbed on the dicarboxylic catalytic system (b_2_). Additionally, the adsorption energy of the second C_2_H_2_ molecule absorbed on the dicarboxylic catalytic system (b_3_) was −1.19 kcal·mol^−1^.

**Figure 9:**
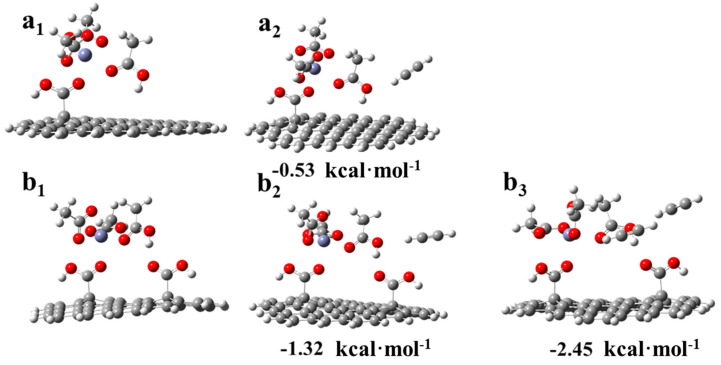
DFT results of the adsorption of acetylene during the reaction: (**a_1_**) acetic acid adsorbed on the reaction site of the monocarboxylic catalytic system. (**a_2_**) Adsorption of acetylene during the reaction in the monocarboxylic catalytic system. (**b_1_**) Acetic acid adsorbed on the reaction site of the dicarboxylic catalytic system. (**b_2_**) Adsorption of the first acetylene molecule during the reaction in the dicarboxylic catalytic system. (**b_3_)** Adsorption of the second acetylene molecule during the reaction in the dicarboxylic catalytic system.

These results revealed that the existence of a dicarboxylic system reduced the adsorption strength of C_2_H_2_ on the catalysts, and this corresponded to the TPD results (Figure 7a). Additionally, to correlate the actual experimental process to the possible mechanism [8,9,11], a hypothetical model was developed for the adsorption of acetylene during the reaction. As shown in Figure 9(a_1_), a molecule of CH_3_COOH was first adsorbed on the monocarboxylic catalyst to form a complex. Subsequently, C_2_H_2_ was adsorbed on the catalyst and reacted with an acetate molecule in the complex (Figure 9(a_2_)), and the adsorption energy was −0.5315 kcal·mol^−1^, which was a tremendous reduction compared with the adsorption energy in Figure 8(c_2_). Thus, the absorption of C_2_H_2_ by the active sites in the reaction process was inhibited by the adsorbed CH_3_COOH molecule. As a rate-limiting step, the rapid decrease in the adsorption strength of C_2_H_2_ exerted a negative effect. However, regarding the dicarboxylic catalyst, the existence of the supererogatory –COOH (–COOH on or near the surface) boosted the adsorption energy of C_2_H_2_ on the reaction site during the reaction from −0.531 (Figure 9(a_2_)) to −1.32 kcal·mol^−1^ (Figure 9(b_2_)), and the adsorption energy of the second C_2_H_2_ molecule was −2.45 kcal·mol^−1^ (Figure 9(b_3_)). The results indicated that the enhancing C_2_H_2_ adsorption strength in the rate-limiting step in the actual experimental process effectively accelerated the reaction rate. Thus, Zn/AC-30 exhibited high performance.

## 4. Conclusions

Summarily, a series of zinc-based catalysts with abundant OCGs were prepared by AC-x (x = 10, 20, 30, 45, and 60), which were obtained by simple thermal treatment, as high-performance catalysts for the acetoxylation of acetylene. The OCGs contents were regulated by different ozone treatment durations (10, 20, 30, 45, and 60 min). The content ratios of –COOH and –OH correlated positively with the catalytic performance by changing the adsorption strengths and capacities of C_2_H_2_ and CH_3_COOH. Further experiments and the DFT results demonstrated that additional –COOH in the dicarboxylic catalytic system would significantly improve the adsorption capacity of the active center for acetylene during the reaction. The increase in the adsorption rate of C_2_H_2_ in the rate-limiting step promoted the reaction rate, thereby proving its high performance. Such work would attract increased attention toward revealing the role of OCGs in the acetylene acetoxylation reaction, and thus further industrial catalytic applications, thereby affording a new strategy for the design and synthesis of highly efficient catalysts.

## Figures and Tables

**Table 1:** Pore structures of the supports before and after ozone treatments at different durations.

	BET Surface Area m^2^·g^−1^	Pore Volume cm^3^·g^−1^	Pore Volume (1.7–300 nm)	Pore Diameter nm
AC	1083	0.565	0.135	2.08
AC-10	1039	0.541	0.112	2.08
AC-20	1000	0.521	0.105	2.08
AC-30	974.6	0.508	0.105	2.08
AC-60	922.7	0.483	0.0993	2.09

**Table 2:** Total oxygen and different OCGs contents of the different supports as calculated by XPS analysis and the Boehm titration method.

	XPS				Boehm Titration Method *		
	O Contents Atomic%	C=O	C–OH/C–O–C	COOH	O Contents Atomic%	OH	COOH
**AC**	5.48	1.96	2.37	1.15	5.10	1.58	1.22
**AC-10**	8.51	2.71	3.82	1.96	7.34	1.84	3.32
**AC-30**	9.79	3.03	4.19	2.57	10.10	2.60	6.48
**AC-60**	10.94	3.54	4.84	2.46	11.37	4.23	6.38

* The proportion of –OH and –COOH was speculated by experimental data.

## Data Availability

The data presented in this manuscript are available on request from the corresponding author.

## Appendix A

The Boehm titration method: Use separate 25 mL NaHCO_3_ (0.1 mol·L^−1^), Na_2_CO_3_ (0.1 mol·L^−1^) and NaOH (0.1 mol·L^−1^) solutions. Mix with the 0.1 g AC-x sample and oscillate for 30 min. Leave undisturbed at room temperature for 24 h then filter. Filtrates are titrated with 0.1 mol·L^−1^ HCl solution. The content of OCGs can be obtained by measuring the difference in the amount of neutralization of the three reagents.

## Appendix B

TPD analysis of C_2_H_2_ and CH_3_COOH: First, the supports and catalysts to be tested were adsorbed C_2_H_2_ and CH_3_COOH under the actual reaction conditions (reaction temperature: 493 K) in the reaction tube. After 4 h adsorption with C_2_H_2_ and CH_3_COOH, the obtained products were tested by TPD. TPD measurements were conducted by heating the products from 353 to 1073 K at a 10 K·min^−1^ heating rate in an argon atmosphere.
